# Whole-genome sequencing provides new insights into genetic mechanisms of tropical adaptation in Nellore (*Bos primigenius indicus*)

**DOI:** 10.1038/s41598-020-66272-7

**Published:** 2020-06-10

**Authors:** Gerardo Alves Fernandes Júnior, Henrique Nunes de Oliveira, Roberto Carvalheiro, Diercles Francisco Cardoso, Larissa Fernanda Simielli Fonseca, Ricardo Vieira Ventura, Lucia Galvão de Albuquerque

**Affiliations:** 1 School of Agricultural and Veterinarian Sciences, São Paulo State University (UNESP), Jaboticabal, SP 14884-900 Brazil; 2 School of Veterinary Medicine and Animal Science, University of São Paulo (USP), Pirassununga, SP 13635-900 Brazil

**Keywords:** Genetics, Sequencing, Sequence annotation

## Abstract

Most of the knowledge about genetic variants at the sequence level in cattle is for *Bos primigenius taurus* populations. Here, we presented a complete genomic characterization of 52 Nellore (*Bos primigenius indicus*) bulls, revealing specific zebu DNA variants with putative impact in tropical adaptation and productive traits. Single nucleotide polymorphisms (SNPs) and insertion/deletion (INDELs) mutations were identified using the newest bovine reference genome ARS_UCD1.2, and variant functional consequences were predicted using the Ensembl VEP software. A total of 35,753,707 SNPs and 4,492,636 INDELs were detected and annotated to their functional effects. We identified 400 genes that comprised both, a SNP and an INDEL, of high functional impact on proteins (i.e. variants that cause protein truncation, loss of function or triggering nonsense-mediated decay). Among these, we highlight the following genes: BoLA, associated with cattle immune response to infections and reproduction aspects; HSPA8, DNAJC27, and DNAJC28, involved with thermoregulatory protective mechanisms in mammals; and many olfactory signaling pathway related genes that are important genetic factors in the evolution of mammalian species. All these functional aspects are directly related to cattle adaptability to tropical environments.

## Introduction

Cattle have played important roles in human societies for a long time, supplying products such as milk, meat, leather, and power. The current two major domesticated cattle, taurine (*Bos primigenius taurus*) and indicine or zebu (*Bos primigenius indicus*), descend from the extinct wild aurochs (*Bos primigenius*) that have diverged, between 250,000 and 330,000 years ago, into two distinct lineages^1^. Taurine cattle originated from the domestication of the *Bos primigenius primigenius* at, approximately, 10,000 years ago in the Fertile Crescent, while the indicine cattle descendant of the *Bos primigenius nomadicus*, domesticated about 8,000 years ago in the Indus Valley^1–5^.

Natural selection, followed by the post-domestication selection driven by humans modified cattle genotypes, leading to distinct genetic and phenotypes profiles between taurine and indicine cattle. In general, these two groups have been selected and are adapted to temperate and tropical environments, respectively^2,4^. Since the taurine cattle genome sequencing^6^, an extensive use of re-sequenced animals has allowed the identification of a considerable number of genetic variants segregating in different populations, essentially represented by taurine breeds^7–13^. To this point, a few studies have explored sequence information of indicine cattle breeds^14–16^.

Nellore figures between the most important domestic indicine cattle with a great impact on the global beef industry. It is a tropically adapted breed and the main responsible for transforming Brazil in one of the largest beef producers and exporters of the world^17,18^. The Brazilian Nellore originated from Ongole cattle which were brought to Brazil from India between 1868 and 1963^19^. Crosses with local taurine cattle increased the population size and subsequently backcrosses with the original imported Ongole lineages recovered indicine adaptive and productive traits^20,21^. Indeed, the currently available Brazilian Nellore genetic resources present low levels of taurine introgression^22^. Since the 1950s, well organized Nellore breeding programs have been established in Brazil, in order to explore and improve the adaptability and performance of this breed under tropical environmental conditions^23,24^.

The objectives of the present study were to: 1) characterize the Nellore genomic variability by mapping single nucleotide variants and insertion/deletion mutations based on a complete genome scan of influential Nellore bulls; and 2) unravel specific zebu DNA variants with functional impact in tropical adaptation- and/or production-related traits.

## Results and discussion

The sequencing generated an average of 360 million reads per individual with 97.9% of them being properly paired in the alignment process. The average individual sequence coverage was 18.50-folds (13.08 to 26.26-folds). These results, similar to other re-sequenced reports, which varied from 9 to 30-folds^14,16,25^, indicate the quality of raw sequence data and alignment process as well as that the depth of coverage was adequate for detecting variants with high confidence. The 1000 bull genomes project, for example, requires animals sequenced at a minimum of 10-folds coverage (http://www.1000bullgenomes.com/).

**Figure 1:**
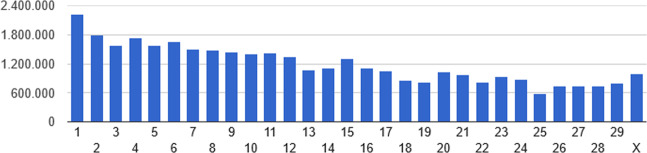
Single nucleotide polymorphism distribution by chromosome.

**Figure 2:**
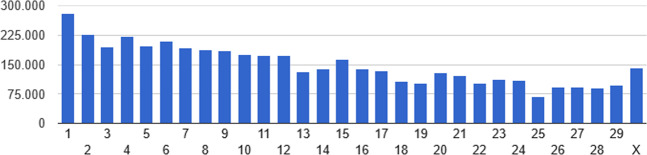
Insertion/deletion distribution by chromosome.

The distribution of the 35,753,707 SNPs and 4,492,636 INDELs across the genome is presented in the Figs. 1 and 2, respectively. Among INDELs, 51.6% and 48.4% were characterized as deletions and insertions, respectively. Most of them (80.0% and 96.7%) correspond respectively to deletions and insertions of less than 3 bp. The prevalence of small INDELs has also been reported in studies involving different breeds^8,14,16^. Here, 13.6% of SNPs and 42.1% of INDELs correspond to novel variants (Ensembl release 96). This high proportion of novel variants, mainly INDELs, suggests Nellore as a unique and significant source of cattle-specific genomic variation, which is in accordance to the known genetic and phenotypic differentiation of this breed in comparison with taurine or even other indicine breeds^4,26–28^. In addition, considering the bovine genome length 2715.85 Mb (https://www.ncbi.nlm.nih.gov/genome/82), on average, there were identified approximately 15 polymorphisms per kb. A similar rate of polymorphism was reported by^25^, who, working with sequencing data of 46 Brahman bulls, reported the identification of about 36 million SNPs and 4.7 million INDELs. All these results are indicative of the magnitude and genetic diversity captured in this group of influential Nellore bulls.

The majority of SNPs and INDELs were intergenic (59.9% and 58,7%, respectively) and intronic (32.3% and 33.4%, respectively) (Table 1). Only 0.3% of the total variants were classified as high or moderate functional impact. It includes the splice acceptor and splice donor, start and stop lost, stop gained, missense, frameshift, inframe insertion or deletion, protein altering variant and transcript ablation variants (Table 1). Even though the small proportion of variants with high or moderate impact on proteins (0.3%), these variants overlapped 17,675 genes (approximately 64% of the total bovine genome annotated genes). As the variants were identified based on a taurine genome reference, this result indicates how expressive is the genetic differentiation with effective impact on phenotypes between Nellore and taurine cattle. To properly address it, our results have to be compared to a database with sequenced animals of various taurine breeds.

**Table 1:** Number of polymorphisms according to the functional class.

Functional classes	Number of SNPs	Number of INDELs
Intergenic_variant	21,410,958	2,636,117
Intron_variant	11,562,722	1,502,267
Upstream_gene_variant	1,230,057	166,009
Downstream_gene_variant	1,067,441	147,044
Synonymous_variant	147,554	—
Missense_variant	115,659	—
3_prime_UTR_variant	116,522	18,607
5_prime_UTR_variant	43,248	5,924
Non_coding_transcript_exon_variant	32,885	3,310
Splice_region_variant	22,554	3,435
Stop_gained	1,975	214
Splice_donor_variant	730	333
Splice_acceptor_variant	476	294
Start_lost	412	13
Stop_lost	244	9
Mature_miRNA_variant	139	25
Stop_retained_variant	128	9
Non_coding_transcript_variant	2	3
Coding_sequence_variant	1	49
Frameshift_variant	—	6,113
Inframe_deletion	—	1,568
Inframe_insertion	—	1,176
Protein_altering_variant	—	107
Transcript_ablation	—	10
Total	35,753,707	4,492,636

**Figure 3:**
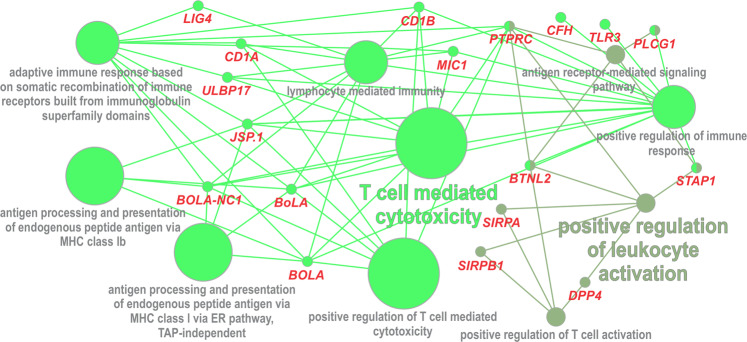
Gene interaction network of genes marked by variants (SNPs and INDELs) with high functional impact.

Here, we identified 400 genes that comprise both, a SNP and an INDEL, of high functional impact on proteins (see Supplementary Table S1). The gene interaction network of this set of genes (Fig. 3 and Supplementary Table S2) showed pathways closely related to the immune response to infections and also impacting reproduction.

The Bovine Major Histocompatibility Complex (MHC), also known as The Bovine Lymphocyte Antigen (*BoLA*), is the primary genetic component of cattle immune system^29^. The main function of *BoLA* class I (MHC-I) is to present peptides to CD8 + T-lymphocytes, which kill virus-infected and neoplastic cells^30^. Bovine lymphocyte antigen restricts cytotoxic cells generated during Theileria infections^31^, evidencing the associations between *BoLA* class l antigens and tick resistance^27^. *BoLA* genes have been flagged as Nellore selection signatures^32^. In addition, non-classical MHC-I genes, such as the *BOLA-NC1*, plays a role in cattle reproduction by regulating maternal immunity to the fetus, which is essential for pregnancy establishment and maintenance^33^, showing that MHC-I play a role in embryo maternal interactions^34^.

As the immune response to infections and reproduction aspects, heat tolerance is one of the main indicatives of adaptability to harsh environments. It is well-documented that zebu animals, such as Nellore, present a more efficient thermoregulatory system to deal with heat stress than taurine breeds^26,35^. This efficiency is expected to be related to several genetically controlled physical and physiological parameters. In this sense, among the genes identified here as potential candidates to tropical cattle adaptability, we could highlight the *HSPA8*, *DNAJC24*, and *DNAJC28*. *HSPA8* plays role in thermoregulatory protective mechanisms in cattle and buffalo under tropical environments^36^, and it is also associated with cellular response to heat stress in goats^37^. *DNAJC24* and *DNAJC28* are type III hsp40 genes that belong to the heat shock protein family^38^. In general, the expression of HSP genes is induced by high temperature, hypoxia, infection and a number of other stresses^30,39^. Thus, zebu-specific variations affecting HSP-mediated response to environmental stressors would explain part of tropical cattle adaptability.

Another important result regarding the putative genes related to adaptability in Nellore is the number of olfactory signaling pathway related genes that have been identified (*OR4S1*, *OR4F15*, *OR2G2*, *OR9A4*, *OR2AP1*, *OR51B4*, *OR5R1*, *OR10AD1, OR2AJ1, OR8U1*, and *OR1P1*). The multigene family of olfactory receptor (OR) is an important genetic factor in the evolution of mammalian species^40^. Variations in cattle OR repertoire could be related to evolution associated with environmental changes^41^. ORs are expressed on millions of olfactory sensory neurons within the olfactory epithelium, but also in organs outside the nasal cavity where they bind to molecules such as nutrients and metabolites^42^. Physiological responses mediated by these chemoreceptors exert a crucial role in animal’s appetite and energy balance regulation, which could affect the feed intake, weight gain, and animal body composition^42^. Olfactory receptors also affect the reproduction in mammals by playing a role in production of germ cells, which are the precursors of gametes^43^. According to^44^, the OR2AP1 gene, identified in this study, is associated with fertility and semen quality of zebu cattle under heat stress conditions. Thus, genetic variations of OR genes could be directly linked to cattle adaptations under extreme environmental conditions.

**Figure 4:**
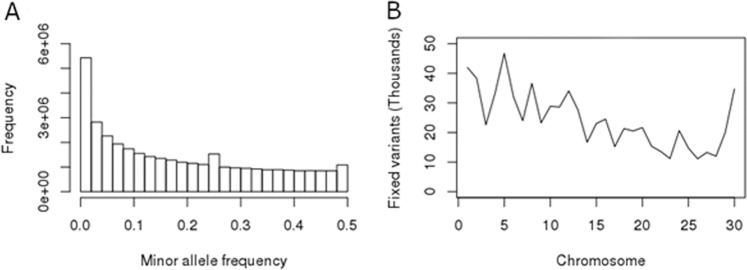
Minor allele frequency (MAF) distribution (**A**) and the number of fixed variants by chromosome (**B**).

We have also evaluated the distribution and annotation of the variants fixed for the alternative allele.^25^ investigated the fixed non-reference alleles in the Brahman genome. According to these authors, such variants could represent genomic regions strongly selected in indicine cattle for providing adaptive advantages in tropical environments. Indeed,^25^ identified some genes with missense mutations fixed in Brahman for the alternative allele related to immunity. Here, the MAF histogram (Fig. 4A) showed a number of fixed variants in Nellore across the genome (Fig. 4B). We have used our Nellore reference population of about 10,000 animals to verify whether these high impact variants fixed for the alternative allele are not really segregating in Nellore. As we can observe in the Supplementary Figure S2, these variants are really rare in our Nellore reference population.

**Figure 5:**
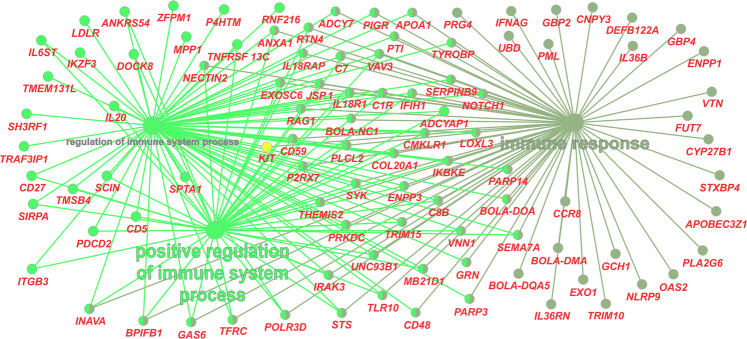
Gene interaction network of genes marked by variants fixed in Nellore with high or moderate functional impact.

Among the Nellore fixed variants, 2,935 have a high or a moderate impact on proteins and they overlap a total of 1,672 annotated genes. As shown in Fig. 5 and Supplementary Table S3, many of these genes play a role in immune system pathways. This result complements our findings discussed before for the list of genes identified as potential candidates to tropical cattle adaptability based on the presence of a simultaneously high impact SNP and INDEL variants. As shown in Fig. 5, this second list of genes (linked to fixed Nellore alleles) also illustrate the possible effective major impact of Bovine Lymphocyte Antigen (BoLA) related genes in zebu cattle adaptation. In addition, a search for known QTLs affecting traits related to adaptation in the QTL database^45^ showed that the region between 15 and 40 Mb on chromosome 23 harbors putative QTLs affecting cattle Cell and antibody-mediated immune response, tick resistance, heat tolerance, and respiratory rate. Nineteen genes associated with missense mutations fixed in Nellore (see Supplementary Table S1), including the genes *BOLA-DQA5*, *BOLA-NC1*, *CNPY3*, *JSP.1*, *TRIM10*, *TRIM15*, and *UBD* presented at the Fig. 5, are located in this QTL region.

At Fig. 5, it could be also highlighted a group of cluster differentiation (CD) genes (*CD5*, *CD27*, *CD48*, and *CD59*), which are expressed on leukocytes and other cells of the immune system^46^, and a group of Interleukin (IL) superfamily members (*IL6ST*, *IL20*, *IL18RAP*, *IL18R1*, *IL36B*, *IL36RN*), which are also important for the immune system regulation. Both CD and IL family genes act on the immune system activation in response to environmental stress, being, in this way, important candidate genes to affect tropical adaptation^47^. Evaluating gene expression patterns in cattle selected for resistance or susceptibility to intestinal nematodes,^48^ found that the *CD27*, *CD45*, and *IL18* genes were highly expressed in resistant animals while the *CD59* and *IL6* were highly expressed in susceptible animals.

Interestingly, as in the list of candidate genes identified based on the presence of a simultaneously high impact SNP and INDEL variants, members of the multigene family of olfactory receptor (OR) genes (*OR11H7*, *OR11L1*, *OR1L1*, *OR2T11*, *OR4D5*, *OR4K14*, *OR51B4*, *OR52M1*, *OR6V1*, and *OR6Y1*) have also been found in the list of genes affected by high or moderate impact fixed Nellore alleles (see Supplementary Table S1). As discussed previously, it has been documented that OR genes are important genetic factors influencing evolution and adaptation of mammalian species^40^.

Beef cattle production in tropical conditions is dominated by Zebu cattle on grass-fed systems, in which the animals are exposed to natural infestations of parasites and have also to tolerate high temperatures and humidity^24^. Remarkably, animal adaptation to the tropics is directly related to its ability to survive, grow and reproduce in the presence of endemic environmental stressors^49^. Our findings have evidenced the polygenic nature of climatic adaptation in the Zebu breed, Nellore, but also suggested the Bovine Major Histocompatibility Complex (BoLA) gene family as one of the main responsible for Nellore adaptation, together with the cluster of differentiation (CD) and Interleukin (IL) superfamily members, and the olfactory receptor (OR) multigene family. The mediated physiological responses by these genes are of paramount importance for the survival and reproduction of animals in challenging environments.

## Conclusions

A genomic characterization of Nellore was done at a sequence level, and new insights into genetic basis of zebu cattle adaptation to harsh tropical environments were provided. We identified various single nucleotide variants, including those that are fixed in this Nellore reference population, and insertion/deletion mutations with high impact in product of genes with functions directly related to cattle adaptability such as disease resistance, heat tolerance, and reproduction.

## Methods

### Animal ethics statement

The animal DNA samples used in this study were extracted from commercially collected semen straws purchased from AI (Artificial insemination) companies or donated to the project.

### Genome sequence information

The studied animals were chosen based on their contributions to the genetic diversity of the Brazilian Nellore population, evaluated through a pedigree file contained 2,688,124 individuals in total, 9,811 (6,040 founders) sires and 915,371 dams. For the sequencing, there were prioritized the founders less genetically related to each other, with high number of progenies, and also taking into account their genetic contributions to our genotyping database, which includes more than 10,000 genotyped animals from five Brazilian Nellore breeding programs (DeltaGen http://deltagen.com.br/, Nelore Qualitas https://qualitas.agr.br/, Cia do Melhoramento, CRV PAINT https://www2.crvlagoa.com.br/paint, and Instituto de Zootecnia http://www.iz.sp.gov.br/). Supplementary Fig. S1 shows the genetic structure of the sequenced sires and how they represent the genetic diversity of the aforementioned Nellore reference population.

The whole-genome sequencing of 52 key Nellore bulls was performed using the Illumina HiSeq X™ Ten platform. Quality control, alignment, and variant calling processes were carried out according to the guidelines suggested by the 1000 bull Genomes Project, available at http://www.1000bullgenomes.com/doco/1000bullsGATK3.8pipelineSpecifications_Run8_Revision_20191101.docx. Initially, the FastQC program^50^ was used to check the raw sequence quality. Trimmomatic software^51^ was used to trim paired and single-reads of the adapter and low-quality bases at their extremities, then to filter out reads that were left with less length than 35 bp or mean qscore lower than 20. The reads were then aligned to the ARS-UCD1.2 reference genome (https://www.ncbi.nlm.nih.gov/assembly/GCA_002263795.2) using the Burrows-Wheeler Aligner - BWA-MEM^52^. Further, BAM files were sorted using SAMtools^53^, and PCR duplicates were removed using the Picard tools (http://broadinstitute.github.io/picard/).

### Variant calling and annotation process

Single nucleotide variants (SNPs) and insertion and deletion mutations (INDELs) were identified using the HaplotypeCaller tool, implemented by the Genome Analysis Toolkit software – GATK^54^. SNPs and INDELs were filtered for quality purposes considering the following exclusion criteria^25^: quality by depth - QD < 2.0; Fisher Strand test - FS > 60.0; root mean square of the mapping quality score - MQ < 40.0; ranked sum test for the distance of alleles from the end of the reads - ReadPosRankSum < −8.0; mapping qualities of reads - MQRankSum < −12.5; and SOR > 3.0. A total of 35,753,707 SNPs and 4,492,636 INDELs of autosomal chromosomes and X were left after this quality control procedure. The Ensembl VEP platform^55^ was then used for variant annotation.

### *In silico* functional analysis of genes comprising variants of high impact on proteins

The Ensembl VEP software classified SNPs and INDELS according to their functional consequences on transcripts. Variants were classified as high when they caused premature stop codons, loss of function or trigger nonsense-mediated decay, and as moderate if lead to non-disruptive variants that might change protein effectiveness. Genes comprising high impact markers were split into two lists: 1) genes containing at least a SNP and an INDEL, both with high functional impact on proteins; and 2) genes marked by high or moderate functional impact variants that are fixed in the sequenced sires. Each list of genes was independently submitted to the ClueGO^56^, a Cytoscape^57^ plug-in that integrates Gene Ontology and KEGG pathways to create an organized GO/pathway annotation network^56^, to verify whether these genes could be statistically (p-value <0.01) related to pathways associated with cattle adaptability in the tropics.

## Supplementary information

Supplementary information.

Supplementary information2.

Supplementary information3.

Supplementary information4.

Supplementary information5.

## Data Availability

The data used in this study were obtained under license and so cannot be publicly available.
