# Author Correction: Whole-genome sequencing provides new insights into genetic mechanisms of tropical adaptation in Nellore (*Bos primigenius indicus*)

**DOI:** 10.1038/s41598-021-81882-5

**Published:** 2021-01-26

**Authors:** Gerardo Alves Fernandes Júnior, Henrique Nunes de Oliveira, Roberto Carvalheiro, Diercles Francisco Cardoso, Larissa Fernanda Simielli Fonseca, Ricardo Vieira Ventura, Lucia Galvão de Albuquerque

**Affiliations:** 1 School of Agricultural and Veterinarian Sciences, São Paulo State University (UNESP), Jaboticabal, SP 14884-900 Brazil; 2 School of Veterinary Medicine and Animal Science, University of São Paulo (USP), Pirassununga, SP 13635-900 Brazil

Correction to: *Scientific Reports* https://doi.org/10.1038/s41598-020-66272-7; published online, 10 June 2020.

The Data Availability section of this Article was incomplete.

“The data used in this study were obtained under license and so cannot be publicly available”.

now reads:

“The data used in this study were obtained under license and so cannot be publicly available. Data are however available from the authors upon reasonable request, and with authorization of Nellore breeding programs.”

