# Supplementary figures and images for: Whole-genome sequencing provides new insights into genetic mechanisms of tropical adaptation in Nellore (Bos primigenius indicus)
Source: Sci Rep. 2020 Jun 10;10:9412. doi: 10.1038/s41598-020-66272-7 (PMC7287098; doi:10.1038/s41598-020-66272-7)

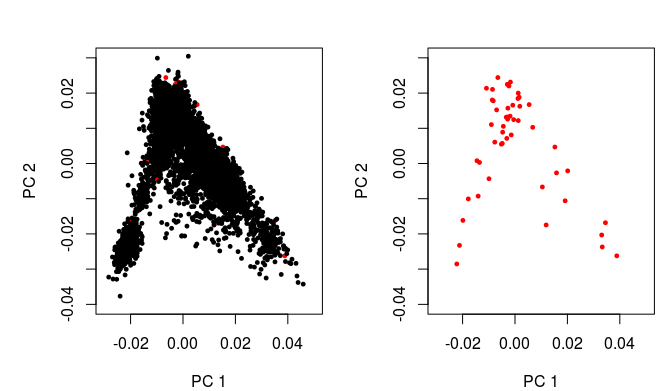

Supplement: Supplementary file 2 — Supplementary information2. [file 41598_2020_66272_MOESM2_ESM.png]

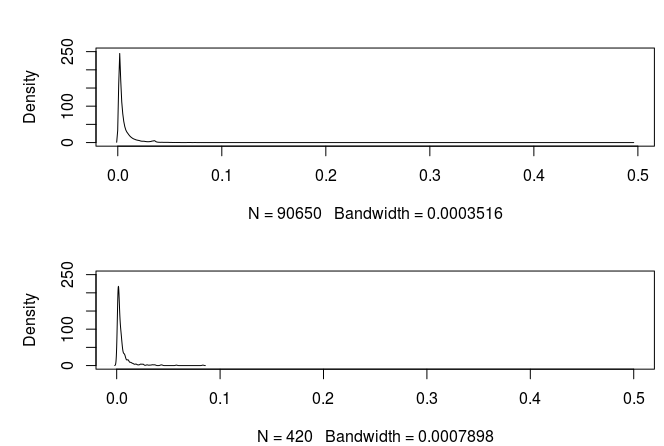

Supplement: Supplementary file 3 — Supplementary information3. [file 41598_2020_66272_MOESM3_ESM.tiff]
